# Temporal trends in the incidence of adverse effects of medical treatment in BRICS countries from 1990 to 2021: an age-period cohort analysis

**DOI:** 10.3389/fpubh.2025.1508272

**Published:** 2025-06-24

**Authors:** Xingmin Wei, Lu Jiang, Zhidong Zhang, Longjian Shang, Kun Liu, Xiaoang Qin, Gaoheng Ding, Lu Liu, Jianjun Wu

**Affiliations:** ^1^School of Public Health, Gansu University of Chinese Medicine, Lanzhou, China; ^2^The Collaborative Innovation Center for Prevention and Control by Chinese Medicine on Diseases Related Northwestern Environment and Nutrition, Lanzhou, China; ^3^Department of Epidemiology, School of Public Health, Air Force Medical University, Xi’an, China

**Keywords:** incidence, age-period-cohort, trend, AEMT, BRICS

## Abstract

**Background:**

Significant disability-adjusted life year (DALY) losses are caused annually by adverse effects of medical treatment (AEMT), a serious public health concern worldwide that continuously strains nations’ socioeconomic progress. As they account for more than half of the world’s population and exhibit notable variation in healthcare resource distribution, the BRICS nations—Brazil, Russia, India, China, and South Africa—have emerged as a crucial observational cohort for researching healthcare safety issues. This study evaluated trends in the incidence of AEMT across BRICS nations from 1990 to 2021.

**Methods:**

This study evaluated trends in the incidence of AEMT in the BRICS nations between 1990 and 2021, utilizing data from the Global Burden of Disease (GBD) 2021 database. We employed Joinpoint regression to determine the average annual percentage change (AAPC) in incidence. Additionally, net drift, localized drift, age-specific curves, and period/cohort relative risk were estimated through an age-period-cohort (APC) model implementing the intrinsic estimator (IE) algorithm.

**Results:**

Between 1990 and 2021, the incidence of AEMT decreased in both South Africa and China. Notably, China exhibited a more pronounced decline, with an AAPC of −1.30% (from 36.94 per 100,000 to 24.65 per 100,000), compared to South Africa’s AAPC of −0.98% (from 117.82 per 100,000 to 86.57 per 100,000). In contrast, Brazil and Russia showed upward trends. Brazil experienced the most substantial increase, rising from 23.06 per 100,000 to 75.09 per 100,000 (AAPC +3.88%), while Russia’s incidence grew less markedly, from 153.97 to 188.67 cases per 100,000 (AAPC +0.65%). Notably, China exhibited a consistently lower incidence of AEMT compared to other BRICS nations. The burden of AEMT disproportionately affected the older adult population in Brazil, South Africa, India, and Russia, whereas in China, the highest incidence was observed among newborns and young children. Regarding cohort risk, all nations demonstrated a reduction over time, except for Brazil and Russia, where cohort relative risk increased significantly.

**Conclusion:**

Over the past three decades, divergent trends in AEMT incidence have been observed across the BRICS nations. To strengthen AEMT prevention, these countries should prioritize optimizing existing healthcare resources, such as workforce training and surveillance systems. Additionally, targeted interventions—including enhanced care for vulnerable populations (e.g., young children, preschoolers, and the older adult)—are critical to further reducing AEMT incidence.

## Introduction

Adverse effects of medical treatment (AEMT) are defined as injuries resulting from surgical procedures, medication errors, healthcare-associated infections/allergic reactions, or negligent/substandard care, which severely undermine patient safety and hospital care quality (1). Despite the World Health Organization (WHO) designating “avoidance of medical harm” as a primary goal of its global patient safety initiatives, AEMT remain prevalent. Globally, an estimated 421 million hospitalizations and 42.7 million AEMT occur annually, leading to 23 million disability-adjusted life years (DALYs) lost each year. Notably, approximately two-thirds of these events are concentrated in low- and middle-income countries (LMICs) (2). According to a landmark report by the Institute of Medicine (IOM) (3), medical errors in the United States are estimated to result in over 98,000 deaths annually, with direct healthcare costs related to AEMT amounting to $1 trillion. Although these findings have intensified concerns about the long-term safety of medical interventions, most existing research disproportionately focuses on high-income countries. A systematic analysis of AEMT trends and their determinants in emerging economies, particularly BRICS nations, remains critically understudied.

Home to over 50% of the global population, the BRICS nations collectively represent the world’s most populous bloc of developing economies (4). Consequently, their healthcare systems, demographic structures, and disease burdens diverge markedly from those of high-income countries. For example, the American Academy of Pediatrics (AAP) reports that while the United States maintains a 3:1 nurse-to-physician ratio, China’s ratio stands at 1:1. Furthermore, the United States employs five times more nurses per capita and nearly twice as many pediatricians per child compared to China, highlighting its significantly more advanced healthcare workforce development systems (5). While the United Kingdom has seen stable age-standardized coronary artery disease (CAD) incidence alongside a dramatic decline in cardiovascular mortality (6), China experienced a rising trend in overall cardiovascular disease prevalence among older adults between 2011 and 2018 (7). This divergence suggests distinct healthcare-associated risk exposure patterns within BRICS nations, potentially driven by variations in prevention strategies and healthcare infrastructure. Long-term trends in the incidence of AEMT across BRICS nations (Brazil, Russia, India, China, and South Africa), as well as age-period-cohort (APC) interactions, remain understudied. This gap stems from the predominant focus of existing research on longitudinal data from high-income countries. Furthermore, systematic analyses of mechanisms linking AEMT incidence to healthcare technology diffusion (such as South Africa’s community health worker [CHW] training programs to strengthen primary care, and China’s nationwide implementation of electronic prescribing systems in tertiary hospitals) or policy interventions (including South Africa’s 2018 establishment of the South African Health Products Regulatory Authority [SAHPRA] to enhance drug approval oversight, and China’s standardized surgical aseptic protocols [SOPs] to improve safety) are critically lacking. By leveraging the APC model and data from the Global Burden of Disease Study 2021 (GBD 2021), this study aims to analyze how age, period, and cohort dynamics shape disease trends. The findings will provide evidence to enhance healthcare quality control in BRICS nations and establish risk prediction frameworks that may inform public health strategies in other LMICs. Ultimately, this work seeks to serve as a reference for early risk identification in LMICs while supporting targeted improvements in BRICS healthcare systems.

## Materials and methods

### Data source

The data used in this study were obtained from the GBD 2021 database, which is a comprehensive database of 369 diseases and health conditions from 204 countries, which is available at https://vizhub.healthdata.org/. We used the International Classification of Diseases and Injuries (ICD-10) to define AEMT, and then we obtained the incidence and age-standardized incidence rates of AEMT for all age groups of males and females in the BRICS countries from 1990 to 2021 from this database. The available data used in this study were anonymized, publicly accessible, and the University of Washington Institutional Review Board approved waiver of informed consent.

### Statistical analysis

Using the WHO world standard population and the direct method, age-standardized rates (ASR), which comprises ASIR, ASDR, as well as ASDALY rate, the formula was:

$\mathrm{ASR}=\frac{\sum_{i=1}^{A}a_{i}w_{i}}{\sum_{i=1}^{A}w_{i}}\times 1$ 00,000.

(The variables *ai* and *wi* denote the ith age class and the total amount of individuals in a single age segment).

#### Joinpoint regression analysis

Using Joinpoint 4.9.0 software, we analyzed the age-standardized incidence rate (ASIR) of AEMT in BRICS nations from 1990 to 2021. We calculated the average annual percentage change (AAPC) and its 95% confidence interval (CI). An upward trend was defined by AAPC values greater than zero, while a downward trend was indicated by AAPC values less than zero.

#### Age-period-cohort model

Given the compatibility of the Poisson distribution with the intrinsic estimator (IE) algorithm for modeling count data such as disease incidence, this study employed the APC model. Disease incidence served as the dependent variable, with age, period, and birth cohort as independent variables. A key strength of this approach lies in the IE method’s ability to resolve parameter uncertainty and covariance inherent in age, period, and cohort effects, thereby isolating their independent contributions. This contrasts with traditional analytical methods, which struggle to disentangle these three temporal dimensions (8). All of the ages in the database are included in the model, and every 5 years, they are split into one age group (0–4 years), 5–9 years, … (90–94 years), for a total of 19 groups; the length of the period-interval is typically equal to the length of the age-interval, and the period is also based on the 5-year period as a one-year group, for a total of 6 groups; the birth cohort is defined by the age of the subject and the date of the event, i.e., cohort = period-age, and the birth cohort is made up of 24 groups. The following equation represents the APC model:

$$Y=\log(M)=\mu +{\text{αage}}_{i}+{\text{βperiod}}_{j}+{\text{γcohort}}_{j}+\varepsilon_{i}$$

*Y = log(M)*, where *Y* represents the logarithm of incidence, *μ* denotes the intercept, *αage_i_* quantifies the age effect, *βperiod_j_* captures the period effect, *γcohort_j_* reflects the cohort effect, and *ε_j_* accounts for random error (9).

The APC model utilizing the IE algorithm generates four key metrics: net drift, local drift, longitudinal age curves, and period/cohort relative risk (RR). Net drift quantifies the overall annual percentage change in AEMT incidence over time; Longitudinal age curves depict age-specific incidence rates within a reference cohort; Local drift represents annual percentage changes stratified by both period and age group; RR values indicate risk magnitude: values exceeding 1 signal elevated AEMT incidence, while values below 1 suggest reduced incidence. Methodologically, we conducted APC analysis via the National Cancer Institute’s APC web tool (10), assessed parameter significance using the Wald chi-square test, and visualized results with Origin software.

## Results

### Trends in the incidence of AEMT

**Figure 1:**
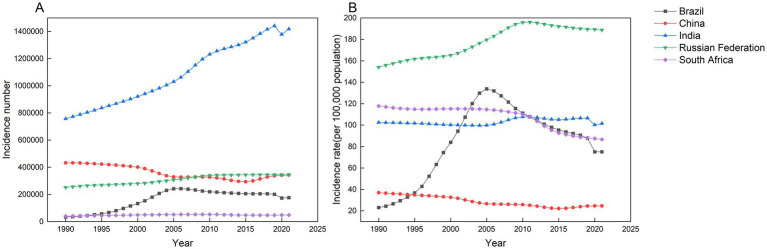
Number of new cases and incidence of AEMT in Brazil, China, India, Russian Federation and South Africa from 1990 to 2021. **(A)** All-age incidence cases. **(B)** Age-standardized incidence rate.

Table 1 and Figure 1 summarize trends in AEMT incidence across BRICS nations. In 2021, India reported the highest incidence cases (14,182,510 cases), followed by Russia (344,751), China (343,388), Brazil (175,841), and South Africa (47,791). Brazil and Russia exhibited sustained increases in incidence from 1990 to 2021: Brazil’s incidence rose from 23.06 per 100,000 in 1990 to 75.09 per 100,000 in 2021 (AAPC +3.88%). Russia’s incidence grew from 153.97 per 100,000 to 188.67 per 100,000 over the same period (AAPC +0.65%). South Africa and China, however, exhibit declining trends in AEMT incidence. South Africa’s incidence decreased from 117.82 per 100,000 in 1990 to 86.57 per 100,000 in 2021 (AAPC: −0.98%). China’s incidence declined more prominently, dropping from 36.94 per 100,000 to 24.65 per 100,000 over the same period (AAPC: −1.30%).

**Table 1:** Characteristics of the incidence of AEMT in BRICS countries, 1990–2021.

Metric		Brazil		China		India		Russia		South Africa	
		1990	2021	1990	2021	1990	2021	1990	2021	1990	2021
Population	Total, *n*	33,476 (26947 to 40807)	175,841 (152129 to 203348)	432,205 (343726 to 531997)	343,388 (277735 to 427062)	757,231 (638117 to 896203)	1,418,251 (1213565 to 1661549)	252,033 (211651 to 302458)	344,751 (292921 to 405575)	40,086 (33576 to 47623)	47,791 (40694 to 56678)
Adverse effects of medical treatment	Incidence rate per 100,000	23.06(18.92 to 27.40)	75.09(64.70 to 87.48)	36.94(30.12 to 44.80)	24.65(19.55 to 31.02)	102.40(87.04 to 120.23)	101.39(87.29 to 117.80)	153.97(130.23 to 186.69)	188.67(161.74 to 220.24)	117.82(100.42 to 138.52)	86.57(74.35 to 101.31)
	AAPC	3.88*(3.31 to 4.45)		−1.30*(−1.34 to −1.26)		−0.08(−0.20 to 0.04)		0.65*(0.62 to 0.68)		−0.98*(−1.04 to −0.93)	

*p < 0.05.

From 1990 to 2021, the incidence of AEMT in China gradually decreased before gradually increasing. Russia mirrored Brazil’s trajectory, demonstrating a distinct phase transition from steep reductions to accelerated growth. In contrast, South Africa sustained a consistent downward trend in incidence, while India showed no statistically significant temporal pattern (Figure 1B). China’s AEMT incidence rate declined steadily from 1990 to 2015, reaching 26.61 per 100,000 by 2015. This downward trend was most pronounced between 2000 and 2005, with the APC registering a significant decrease of 4.22%, corresponding to a reduction from 32.62 per 100,000 in 2000 to 26.61 per 100,000 in 2005. Subsequently, the trajectory reversed between 2015 and 2021. The incidence rose from 22.14 per 100,000 in 2015 to 24.65 per 100,000 in 2021, driven primarily by a 2.74% APC increase during the 2015–2019 period, which marked the steepest upward surge in the observed timeframe.

Brazil exhibited distinct phases in AEMT incidence between 1990 and 2021. From 1990 to 2004, incidence rose steadily, peaking at 129.87 per 100,000 by 2004. The most rapid escalation occurred between 1995 and 1999, marked by a 20.15% APC that propelled the incidence rate from 36.72 per 100,000 to 74.35 per 100,000 within this four-year span. A sustained decline began in 2007, reducing incidence from 127.37 per 100,000 to 75.09 per 100,000 by 2021. This downward trend accelerated markedly between 2019 and 2021, registering an annual decline of 8.18% as incidence dropped from 87.80 per 100,000 to 75.09 per 100,000, marking the steepest reduction observed in the study period.

In Russia, the incidence of AEMT has been steadily increasing since 1990, with the most significant rise occurring between 2001 and 2007. During this period, the APC was 1.98%, resulting in an increase from 167.07 per 100,000 in 2001 to 186.94 per 100,000 in 2007. However, this upward trend reversed in 2010, when incidence rates began to decline. The sharpest decrease occurred between 2010 and 2017, during which the APC dropped to −0.46%, reducing the rate from 196.82 per 100,000 in 2010 to 190.65 per 100,000 in 2017.

The rate of AEMT in South Africa declined steadily from 114.95 per 100,000 in 2004 to 86.57 per 100,000 in 2021. The most pronounced decrease occurred between 2011 and 2014, when the APC reached −4.21%, corresponding to a reduction in incidence from 107.64 to per 100000105.35 per 100,000 during this three-year period.

### Age-specific incidence of AEMT

**Figure 2:**
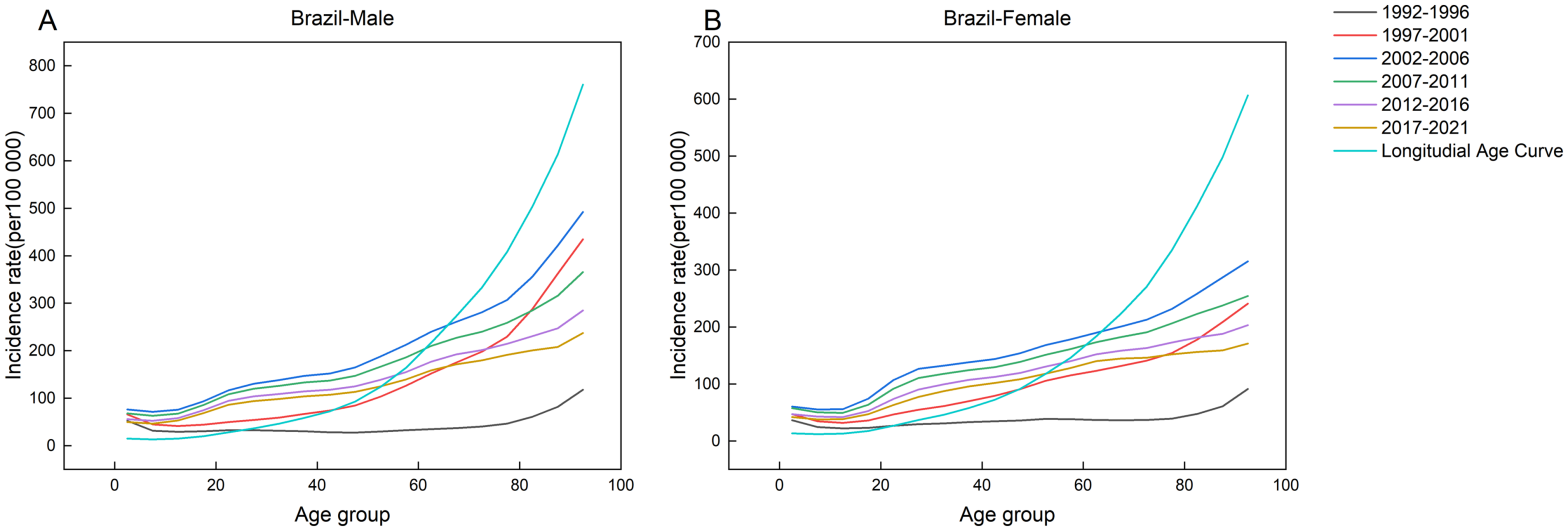
Age-specific incidence of AEMT in Brazil by period, 1990–2021. **(A)** Age-specific incidence rate for males in Brazil. **(B)** Age-specific incidence rate for females in Brazil.

**Figure 3:**
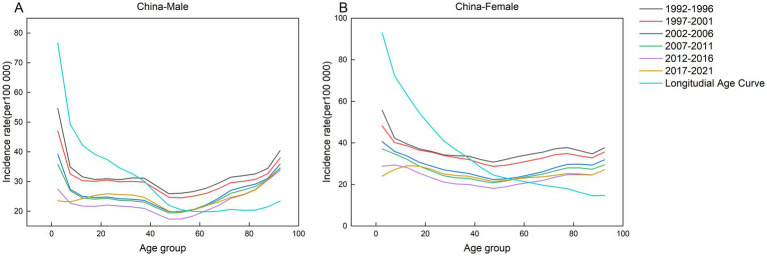
Age-specific incidence of AEMT in China by time period, 1990 to 2021. **(A)** Age-specific incidence rate for males in China. **(B)** Age-specific incidence rate for females in China.

**Figure 4:**
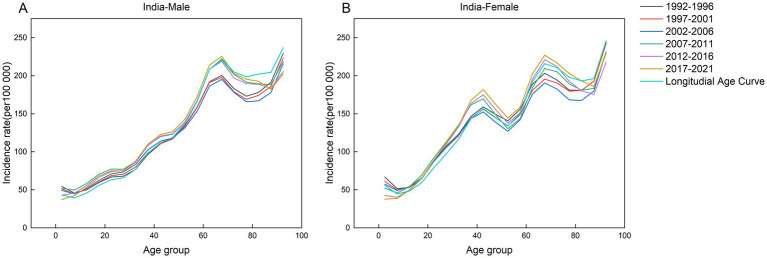
Age-specific incidence of AEMT in India by period, 1990 to 2021. **(A)** Age-specific incidence rate for males in India. **(B)** Age-specific incidence rate for females in India.

**Figure 5:**
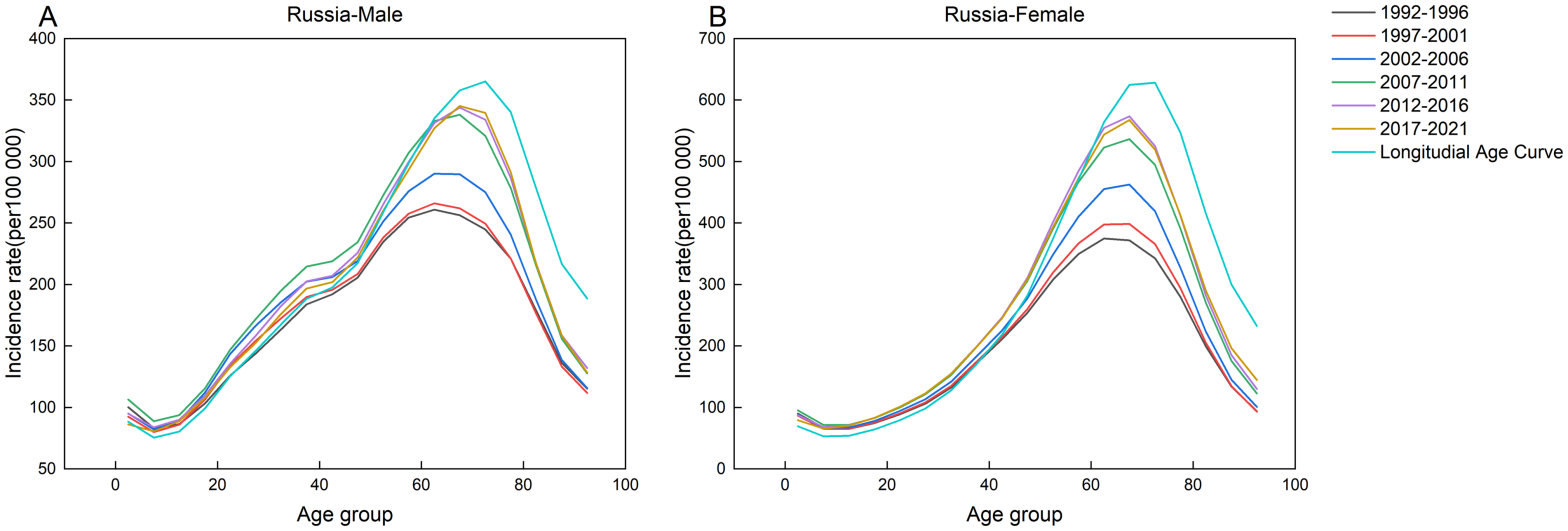
Age-specific incidence of AEMT in Russia by period, 1990 to 2021. **(A)** Age-specific incidence rate for males in Russia. **(B)** Age-specific incidence rate for females in Russia.

**Figure 6:**
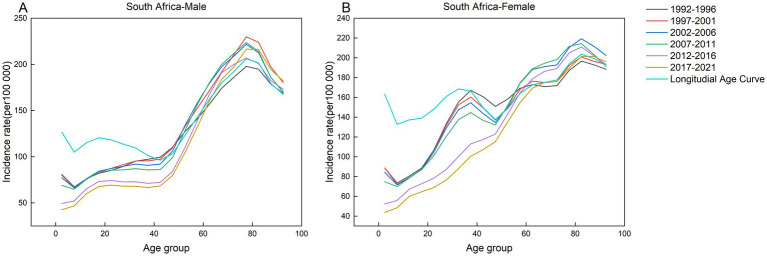
Age-specific incidence of AEMT in South Africa by period, 1990 to 2021. **(A)** Age-specific incidence rate for males in South Africa. **(B)** Age-specific incidence rate for females in South Africa.

This study analyzed the incidence of AEMT across five consecutive five-year intervals (1992–1996, 1997–2001, 2002–2006, 2007–2011, 2012–2016, and 2017–2021) and 19 age cohorts, with the median year of each interval specified (1994, 1999, 2004, 2009, and 2014, respectively). Figures 2–6 present the age-specific patterns of AEMT incidence in BRICS nations from 1990 to 2021. The trend in AEMT incidence among Brazilian women closely mirrors that among men, a pattern also observed in China and Russia. Among Indian populations, the age-specific curves revealed three distinct peaks for females (40–44, 65–69, and 90–94 years), whereas males exhibited only two peaks (65–69 and 90–94 years). In contrast, South Africa demonstrated gender-divergent patterns: incidence peaked at 75–79 years for males and 80–84 years for females, with significant variations across other age groups.

### Net drift and localized drift by age group

**Figure 7:**
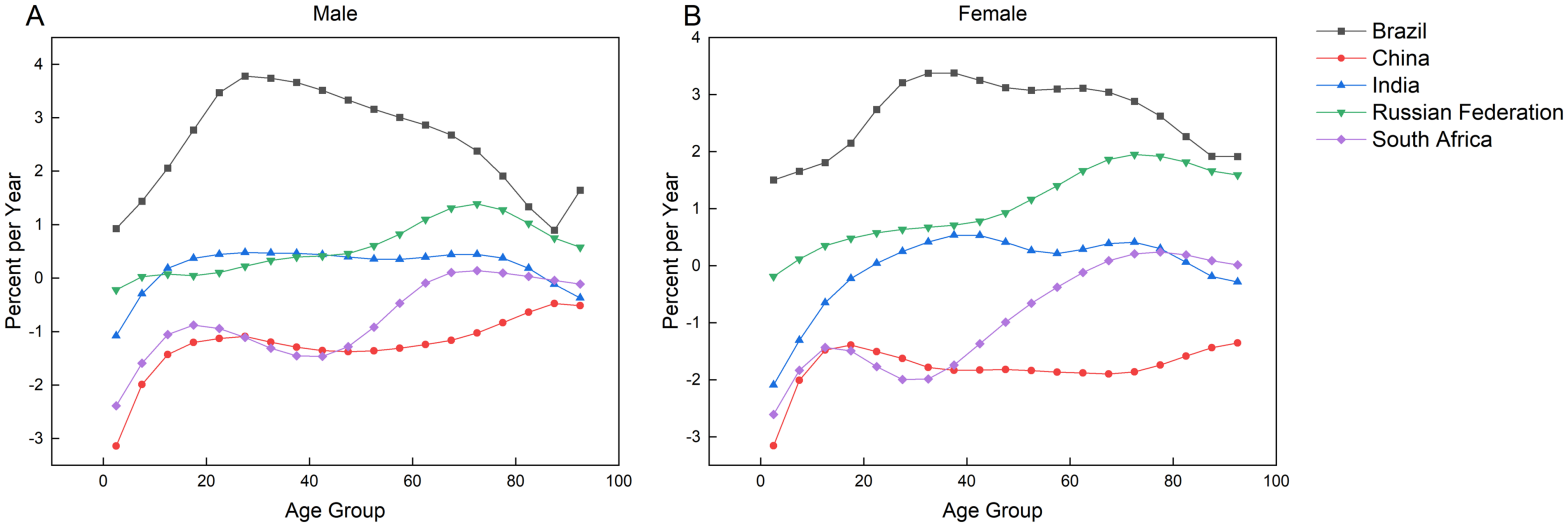
Localized and net drift in the incidence of AEMT in Brazil, China, India, Russia, and South Africa, 1990–2021. **(A)** Local drift and net drift for male incidence. **(B)** Local and net drift for female incidence.

As demonstrated in Figure 7, both China and South Africa exhibited negative overall drift values (below zero), indicating a decline in incidence rates. China showed a steeper reduction than South Africa, with sex-specific drift values of −1.23 [95% CI: −1.32 to −1.15] for males and −1.78 [95% CI: −1.87 to −1.69] for females. In contrast, Brazil displayed the highest positive drift values (2.87 [95% CI: 2.39 to 3.35] for males and 2.86 [95% CI: 2.54 to 3.17] for females), reflecting the most rapid acceleration in incidence among all studied countries. Similarly, India and Russia also had positive drift values (above zero), though lower than Brazil’s. Significant gender disparities in annual incidence change were observed between India and Russia. In India, the annual change rate for males was 0.32 (95% CI: 0.25 to 0.39), which was 2.13 times higher than the female rate of 0.15 (95% CI: 0.08 to 0.23). Conversely, Russian females exhibited a substantially elevated rate of 1.09 (95% CI: 1.05 to 1.12), corresponding to 1.82 times the male rate of 0.60 (95% CI: 0.53 to 0.68).

Figure 7 demonstrates that local drift values exceeded zero across most age groups in BRICS nations, indicating an upward trend in AEMT incidence. The most rapid increases were observed among Brazilian males aged 25–29 (3.78) and females aged 35–39 (3.78), followed by males aged 30–34 (3.74) and females aged 30–34 (3.37). While localized drift curves across BRICS nations predominantly exhibit upward trends, notable exceptions exist in specific age groups. In South Africa, the 0–64 years cohort showed negative drift values (males: −2.39 to −0.09; females: −2.61 to −0.12). Similarly, Russia’s 0–4 years age group displayed declines (males: −0.21; females: −0.29). All Chinese age groups maintained negative drift values, contrasting with the broader regional pattern. In India, downward trends were observed in males aged 0–9 years (−1.08 to −0.29) and females aged 0–19 years (−2.10 to −0.23), with additional reductions in the 85–94 years cohort (males: −0.12 to 0.37; females: −0.19). South African males aged 85–94 years also demonstrated minimal decline (−0.04 to 0.11). These exceptions highlight heterogeneity within otherwise rising incidence trajectories across BRICS populations.

### The effect of age-period-cohort on the incidence of AEMT

**Figure 8:**
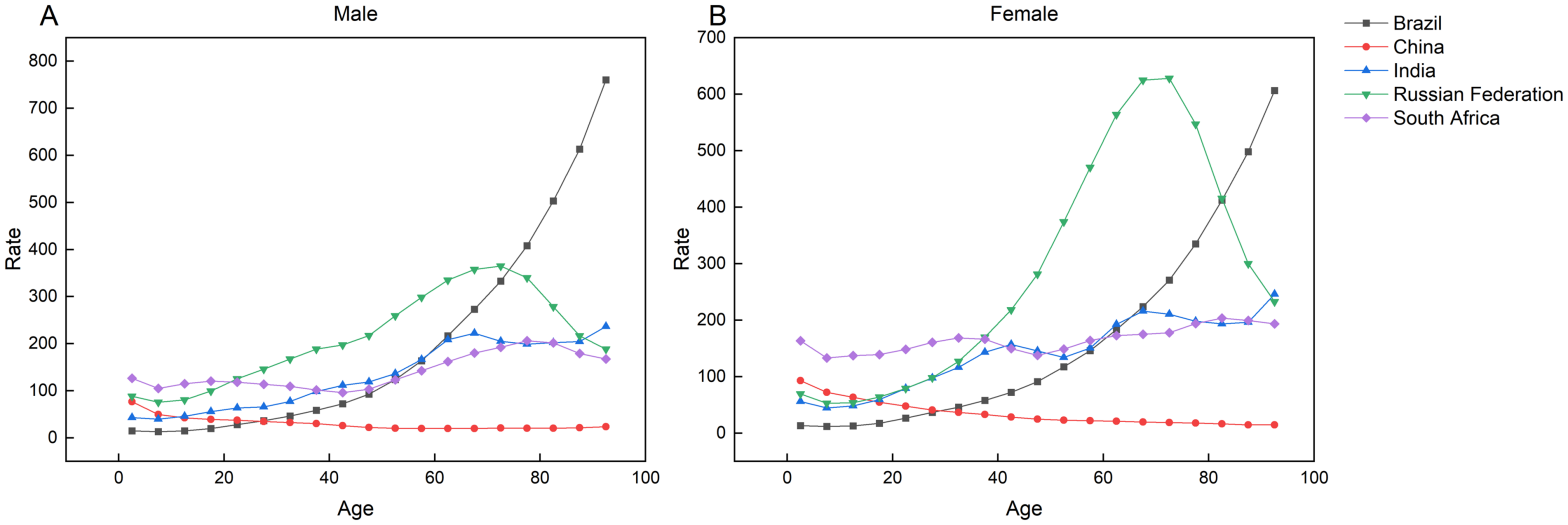
Parameter estimates of the impact of age effects on the incidence of AEMT in Brazil, Russia, India, China and South Africa, 1990 to 2021. **(A)** Age effects in male incidence. **(B)** Age effect of female incidence.

Figure 8 demonstrates distinct age-related patterns in AEMT risk across BRICS nations. In Brazil, both sexes exhibit age-progressive risk escalation, with susceptibility increasing continuously from youth to advanced age. Conversely, China presents an inverse trajectory—peak morbidity risk occurs in the 0–4 year cohort for both genders, followed by progressive risk attenuation with aging. India shows gender-divergent patterns: male incidence peaks modestly in the 65–69 age group, declines until 75–79 years, then resurges to a second peak at 90–94 years; females demonstrate monotonic age-dependent risk accumulation, culminating in maximal incidence at 90–94 years. In South Africa, males exhibit relatively stable incidence rates from birth through the 0–44-year age range, followed by an upward trajectory initiating in the 40–44-year cohort, peaking in the 75–79-year group, and subsequently declining with advancing age. Conversely, females demonstrate a bimodal risk pattern: the highest incidence risk among younger populations occurs in the 30–34-year cohort, while in older age groups (>60 years), risk initially rises before attenuating, ultimately culminating in a distinct peak within the 80–84-year cohort. In contrast, Russia displays a unimodal age-risk distribution for both sexes, characterized by a bell-shaped morbidity curve that peaks sharply in the 70–74-year group before progressively declining with older age.

**Figure 9:**
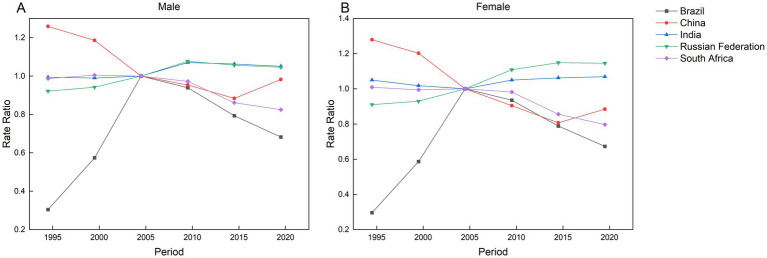
Parameter estimates of the impact of period effects on the incidence of AEMT in Brazil, Russia, India, China and South Africa from 1990 to 2021. **(A)** Period effect of male incidence. **(B)** Period effect of female incidence.

Figure 9 indicates that the incidence risk of AEMT exceeded 1.0 in multiple populations and periods, including Russia (2007 to 2021), China (1992 to 2001), Indian males (2007 to 2021), Indian females across all study periods, South African males (1997 to 2001), and South African females (1992 to 1996), reflecting stagnant or worsening trends in these groups. Conversely, incidence risks below 1.0 in other cohorts suggest a beneficial period effect associated with reduced rates. At the national level, Russia, India, and Brazil exhibited progressive increases in overall incidence risk over time, while China and South Africa demonstrated consistent declines.

**Figure 10:**
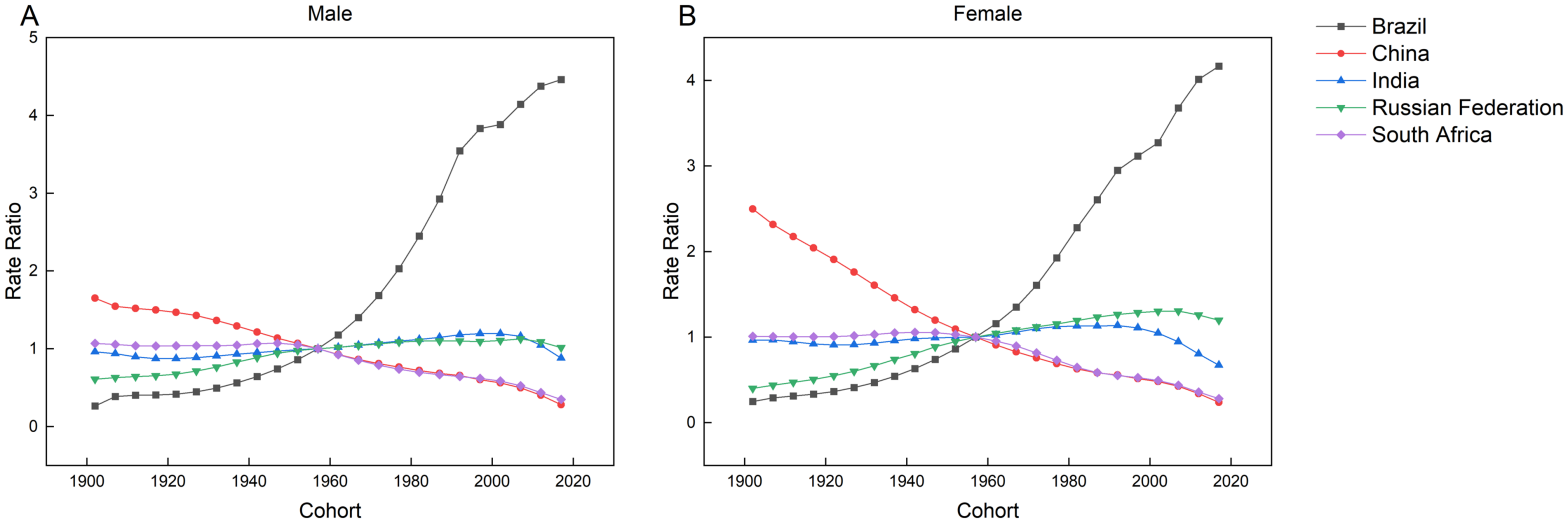
Parameter estimates of the impact of cohort effects on the incidence of AEMT in Brazil, Russia, India, China and South Africa from 1990 to 2021. **(A)** Cohort effect of male incidence. **(B)** Cohort effect of female incidence.

As shown in Figure 10, Brazil and Russia exhibit rising morbidity risks with later birth cohorts, while China and South Africa demonstrate declining trends. In India, males born during the 2000–2004 cohort show the highest risk of AEMT, whereas females born in the 1994–1999 cohort face the peak risk. The overall trajectory in India follows a non-linear pattern: morbidity risk initially escalates with earlier birth cohorts, declines temporarily, and subsequently resurges in more recent generations.

## Discussion

This study identified a declining trend in AEMT incidence between 1990 and 2021 in South Africa and China. South Africa exhibited an AAPC of −0.98%, with incidence decreasing from 117.82 per 100,000 to 86.57 per 100,000, while China showed the steepest reduction (AAPC: −1.30%), declining from 36.94 per 100,000 to 24.65 per 100,000. In contrast, Brazil demonstrated a marked upward trend (AAPC: 3.88%), with incidence surging from 23.06 per 100,000 to 75.09 per 100,000. Even Russia, which had the smallest increase among rising cohorts, recorded a positive AAPC of 0.65%, rising from 153.97 per 100,000 to 188.67 per 100,000. China and South Africa achieved significant progress in reducing AEMT between 1990 and 2021, with China demonstrating the most substantial improvement. In contrast, Russia and Brazil require intensified efforts to mitigate such risks. Notably, China consistently maintained the lowest incidence rate of AEMT among BRICS nations, outperforming Brazil, South Africa, India, and Russia. Furthermore, our analysis identified neonates, young children, and older adult populations as having markedly higher susceptibility to AEMT compared to other age groups. To address these disparities, BRICS countries should develop tailored prevention strategies aligned with their national healthcare contexts, prioritizing high-risk demographic cohorts.

In China, the highest risk of AEMT occurs among children aged 0–4 years, driven by physiological immaturity, underdeveloped disease resistance in infants, and surgical risks linked to developmental variability within this demographic (11). From 1990 to 2021, China achieved sustained reductions in AEMT rates, temporal trends, and generational risks, progress strongly correlated with modernization of its public health infrastructure. China established the National Adverse Drug Reaction Monitoring Center (NADRMC) in 1988 to standardize pharmacovigilance practices. Recent enhancements in regulatory frameworks, including clearly defined reporting protocols and timelines for AEMT, coupled with digital transformation initiatives such as transitioning from manual case reporting to widespread adoption of electronic prescription systems in tertiary hospitals, have significantly improved ADR surveillance and data analytics (12). Supporting this progress, Raccoon et al. demonstrated a measurable decline in anticancer and antibacterial drug related ADR incidence between 2006 and 2016, validating the effectiveness of these systemic improvements (13). In 2009, China implemented the National Essential Medicines Program (NEMP) as a core component of its healthcare reform, aiming to mitigate AEMT through standardized medication protocols and reduced irrational drug use (14). Analysis of data from the National Community Health Services Monitoring Program by Gong et al. revealed measurable post-NEMP improvements: antibiotic prescriptions decreased by 7%, corticosteroid use declined by 1%, and polypharmacy (concurrent use of two or more antibiotics) dropped by 2%, alongside a reduction of 0.2 medications per prescription compared to pre-NEMP implementation periods (15). These findings underscore the program’s efficacy in optimizing prescribing practices. Since the implementation of China’s Medical Quality Management Measures in 2016, hospitals have significantly reduced surgery-associated complications stemming from improper medical device utilization and procedural deviations through two key interventions: (1) establishing standardized operating procedures (e.g., surgical aseptic technique protocols), and (2) implementing rigorous clinical pathway management systems (e.g., disease-specific standardized treatment regimens) (16). These systemic reforms enhanced operational standardization across surgical and therapeutic domains.

The age-specific analysis identified South African males aged 75–79 years and females aged 80–84 years as having the highest morbidity risk. This elevated susceptibility among older adult populations stems from multiple factors: physiological frailty, multimorbidity patterns, medication non-compliance due to inadequate geriatric healthcare guidance, and socioeconomic disparities (17). Concurrently, South Africa achieved a generalized decline in AEMT and temporal period effects between 1990 and 2021, a trend attributable to targeted national policy interventions implemented during this timeframe. Established in 2018, the South African Health Products Regulatory Authority (SAHPRA) has enhanced drug approval protocols and postmarket surveillance systems, effectively reducing AEMT risks (18). Concurrently, the government’s Free Primary Healthcare Policy expanded service coverage while training community healthcare workers (CHWs) in essential clinical competencies including wound care and chronic disease management. These community-level interventions decreased unnecessary hospital admissions, thereby lowering nosocomial infection rates and iatrogenic errors (19). Our analysis revealed reduced incidence risks of AEMT among South African birth cohorts, specifically males born post-1947 and females post-1942. This trend suggests that historical public health interventions implemented in South Africa may have contributed to mitigating such risks through systemic healthcare improvements.

India exhibits an age-related risk profile comparable to South Africa’s, with peak morbidity risk concentrated in the 90–94-year age cohort. Furthermore, AEMT incidence and temporal period effects demonstrated sustained escalation across India from 1990 to 2021. This trend may be partially explained by critical healthcare workforce shortages – particularly in eastern states where health worker density reaches only 20 per 10,000 population, below the WHO minimum threshold of 22.8 per 10,000 (20). Such workforce deficits correlate strongly with elevated risks of medication errors and clinical procedural deviations, as evidenced by increased iatrogenic incident reports in low-coverage regions (21). Studies reveal that 70% of India’s healthcare services are sourced from private hospitals, reflecting a comparatively more developed private sector relative to other BRICS nations (22). However, these facilities frequently operate under inadequate regulatory oversight and exhibit systemic overprescription practices, leading to elevated rates of iatrogenic errors and medication-related adverse events (23). Notably, India’s AEMT is increasing twice as rapidly among males compared to females — a trend diverging from BRICS counterparts. This gender disparity may be linked to higher male engagement in risk behaviors such as tobacco use, alcohol consumption, and other modifiable health risk factors. Extensive research confirms that smoking exerts detrimental effects on human health while amplifying susceptibility to AEMT. India ranks as the world’s second-largest tobacco producer and consumer, second only to China (24). Notably, analysis of Indian birth cohorts reveals promising trends: individuals born after 2002 (males) and 1992 (females) demonstrate marked reductions in AEMT risks. These findings suggest measurable progress in India’s pharmacological safety protocols and AEMT mitigation strategies. Sustained implementation of tobacco control measures combined with declining youth tobacco adoption rates could significantly mitigate India’s burden of treatment-related complications in coming decades.

Russian males and females exhibit a unimodal age-specific morbidity risk pattern, peaking in the 70–74 year cohort before declining with advancing age. This pattern coincides with systemic barriers to primary healthcare access in Russia, evidenced by higher out-of-pocket expenditures and reduced public health funding relative to the WHO European Region average (25). These systemic challenges likely exacerbate the elevated risk of AEMT observed in the population. Russia’s healthcare infrastructure faces systemic challenges. According to the Russian Ministry of Health, between 2005 and 2018, central regions experienced a 60% reduction in hospital numbers alongside a decline in rural bed capacity from 49.6 to 38.8% (26). This resource depletion amplifies the burden of treatment-related complications. Furthermore, Russia’s extreme continental climate exacerbates public health risks through two distinct mechanisms: (1) prolonged indoor congregation during subzero temperatures facilitates airborne transmission of influenza and tuberculosis in poorly ventilated spaces; (2) heightened antibiotic misuse driven by recurrent respiratory infections increases nosocomial infection risks (27). Russia exhibits a gender disparity in AEMT incidence, with women experiencing rates 1.8 times higher than men – a pattern contrasting sharply with India’s epidemiological profile. This discrepancy may stem from multifactorial risks: (1) heightened perioperative vulnerability in women linked to physiological differences, (2) elevated obstetric complications during pregnancy/delivery. Notably, Russia’s most recent birth cohorts demonstrate declining AEMT risks, a trend potentially attributable to nationwide healthcare digitization initiatives since 2009. The expansion of electronic health record systems has enhanced care coordination and treatment standardization, thereby reducing iatrogenic errors and improving pharmacological safety outcomes (28).

Our analysis reveals an age-progressive risk escalation for AEMT among both Brazilian males and females. Brazil demonstrates significantly higher incidence rates of AEMT compared to other BRICS nations, characterized by sustained upward trajectories in temporal period effects and birth cohort risks from 1990 to 2021. Notably, Brazil consistently maintained the highest incidence rates within the BRICS grouping throughout this timeframe. Brazil, like many developing nations, faces significant constraints in healthcare resource allocation. These systemic limitations contribute to suboptimal hand hygiene compliance rates among medical staff (≤50% in public hospitals), directly exacerbating the spread of multidrug-resistant (MDR) bacterial infections (29). This deficit in infection control protocols amplifies healthcare-associated infection risks, particularly in resource-limited public healthcare settings (30). Crucially, evidence from Finland demonstrates that sustained hand hygiene adherence above 80% compliance over 24 months correlates with measurable reductions in nosocomial infection rates (31), highlighting actionable thresholds for Brazil’s healthcare reform priorities. Population aging in Brazil elevates the risk of AEMT through multiple pathways: older adult patients often present with multiple comorbidities, reduced physical function, and diminished tolerance to surgical interventions and pharmacological treatments. This demographic shift is compounded by accelerated population aging trends – the proportion of Brazilians aged 60+ is projected to rise from 9.7% (2004) to 13.7% (2014), 18.6% (2030), and 33.7% (2060) of the total population (32). These intersecting factors create a self-reinforcing cycle where aging physiology and expanding geriatric populations synergistically increase systemic healthcare risks.

## Conclusion

Over recent decades, China and South Africa have demonstrated sustained declines in AEMT incidence, with China achieving particularly notable reductions while maintaining consistently low rates – evidence of effective national control strategies. In contrast, Brazil, India, and Russia continue to experience rising AEMT incidence. This disparity underscores the urgent need for optimized healthcare resource distribution and enhanced policy interventions targeting systemic healthcare quality improvements across developing economies. Newborns, preschool-aged children, and older adult populations exhibit significantly higher incidence rates of AEMT compared to other age groups, establishing these cohorts as priority targets for intervention. Consequently, BRICS nations should prioritize three strategic imperatives: (1) implementing age-specific health policies tailored to high-risk demographics, (2) developing tiered early warning systems aligned with epidemiological risk stratification, and (3) establishing precision prevention frameworks based on national healthcare capacity assessments. These measures must account for developmental disparities between member states while addressing systemic vulnerabilities through coordinated surveillance of medical error patterns and optimized resource allocation across prevention, diagnosis, and treatment pathways.

## Data Availability

The original contributions presented in the study are included in the article/supplementary material, further inquiries can be directed to the corresponding author.
